# Prognostic Value of Combined Carbohydrate Antigen 19-9 and Duke Pancreatic Monoclonal Antigen Type 2 Assessment in Biliary Tract Cancer

**DOI:** 10.1245/s10434-026-19488-2

**Published:** 2026-03-26

**Authors:** Ryuta Shintakuya, Kenichiro Uemura, Tatsuaki Sumiyoshi, Kenjiro Okada, Takumi Harada, Sho Tazuma, Yasutaka Ishii, Shiro Oka, Yoshiaki Murakami, Shinya Takahashi

**Affiliations:** 1 Department of Surgery, Graduate School of Biomedical and Health Science, Hiroshima University, Hiroshima, Japan; 2 Department of Gastroenterology and Metabolism, Graduate School of Biomedical and Health Science, Hiroshima University, Hiroshima, Japan; 3 Digestive Disease Center, Hiroshima Memorial Hospital, Hiroshima, Japan

**Keywords:** Biliary tract cancer, Carbohydrate antigen 19-9, Duke pancreatic monoclonal antigen type 2

## Abstract

**Background:**

Carbohydrate antigen 19-9 (CA19-9) represents an established prognostic biomarker for biliary tract cancers (BTCs). Clinical utility remains limited among patients with Lewis antigen negativity or incomplete fucosyltransferase 3 (*FUT3*) gene function, conditions associated with absent or reduced CA19-9 production. Duke pancreatic monoclonal antigen type 2 (DUPAN-2), a biosynthetic precursor of CA19-9, shows compensatory elevation under such conditions. This study evaluated the prognostic significance of the combined preoperative assessment of CA19-9 and DUPAN-2 among patients with resectable BTCs.

**Patients and Methods:**

Patients who underwent curative-intent surgery for BTCs between 2009 and 2022 were included. Eligible patients were classified into four groups according to preoperative biomarker levels: normal CA19-9/normal DUPAN-2 (N/N), normal CA19-9/high DUPAN-2 (N/H), high CA19-9/normal DUPAN-2 (H/N), and high CA19-9/high DUPAN-2 (H/H). Clinicopathological features, overall survival (OS), and recurrence-free survival (RFS) were compared. Multivariate analyses identified independent prognostic factors.

**Results:**

The cohort included 276 patients with BTCs who underwent curative resection, comprising 41 intrahepatic cholangiocarcinomas, 72 hilar cholangiocarcinomas, 57 distal cholangiocarcinomas, 53 gallbladder carcinomas, and 53 ampullary carcinomas. Age and tumor distribution showed no significant intergroup differences. The N/N group demonstrated significantly superior OS and RFS (*P* < 0.001). Multivariate analysis identified the N/N profile as an independent favorable prognostic factor for OS and RFS (*P* < 0.001).

**Conclusions:**

Concurrent normal CA19-9 and DUPAN-2 levels independently predicted favorable outcomes. Combined preoperative biomarker assessment may contribute to prognostic stratification of BTCs, including among patients with Lewis antigen negativity or impaired FUT3 function.

Biliary tract cancer (BTC) represents an aggressive, high-risk malignancy associated with poor prognosis, and surgical resection remains the only curative treatment associated with improved outcomes. Carbohydrate antigen 19-9 (CA19-9) has been reported as a useful biomarker for prognostic evaluation in patients with BTC.^1,2^ Among patients with fucosyltransferase 3 (*FUT3*) gene deficiency, defined as Lewis antigen negativity, or incomplete *FUT3* gene function, CA19-9 production remains absent or markedly reduced, with reported reductions of up to 50%.^3–7^ Duke pancreatic monoclonal antigen type 2 (DUPAN-2), a precursor of CA19-9, undergoes conversion to CA19-9 through FUT3 activity, followed by conversion of CA19-9 to sialyl-Lewis b through FUT2. CA19-9 and DUPAN-2 exhibit a complementary relationship governed by FUT gene expression.^4^ Variants of the FUT3 gene include fully functional (wild type), nonfunctional (null type), and incompletely functional (heterozygous type) forms.^7^ Approximately 40% of patients possess a fully functional FUT3 gene and demonstrate adequate CA19-9 production without DUPAN-2 accumulation. In contrast, approximately 5–18% of patients carry a nonfunctional FUT3 gene, resulting in absent CA19-9 production, defined as CA19-9 levels ≤ 2 U/mL, with compensatory DUPAN-2 accumulation.^5,7,8^ An additional 40% of patients exhibit incompletely functional FUT3 variants, characterized by markedly reduced CA19-9 production and mild DUPAN-2 elevation.^7^ Therefore, interpretation of CA19-9 levels requires consideration of both Lewis antigen status and incomplete FUT3 gene function, supporting combined assessment with DUPAN-2 for accurate clinical evaluation. Recently, the combined assessment of CA19-9 and DUPAN-2 has demonstrated value for prognostic evaluation among patients with pancreatic ductal adenocarcinoma (PDAC).^8–10^ However, evidence addressing this combined approach in BTC remains limited. Routine measurement of both biomarkers began at our institution in 2009 for BTC. This study hypothesized that the combined assessment of CA19-9 and DUPAN-2 could serve as a useful tool for prognostic evaluation among patients with BTCs who underwent curative resection. This study aimed to evaluate the prognostic impact of the combined assessment of CA19-9 and DUPAN-2 in patients with resectable BTCs.

## Patients and Methods

### Study Design

Clinical data were collected through a retrospective review of prospectively maintained institutional databases. BTCs comprised five types: intrahepatic cholangiocarcinoma (ICC), hilar cholangiocarcinoma (HC), distal cholangiocarcinoma (DC), gall bladder carcinoma (GBC), and ampullary carcinoma (AC). Patients who underwent curative-intent surgery for BTCs at the Department of Surgery, Hiroshima University Hospital, between April 2009 and December 2022 were included. Pathological confirmation of BTC was obtained in all cases. Two experienced pathologists specializing in biliopancreatic malignancies independently confirmed each diagnosis using surgically resected specimens. Tumor stage and lymph node metastasis status were classified according to the 8th Edition of the Union for International Cancer Control (UICC) Tumor, Node, Metastasis classification system. This study was approved by the Institutional Review Board of Hiroshima University Hospital (Identification No. E2024-0269). All procedures were performed in accordance with the ethical standards of the 1964 Declaration of Helsinki and its later amendments or comparable ethical standards. Additionally, written informed consent was obtained from all patients before study participation.

### Definition

Normal serum CA19-9 level was defined as ≤ 37 U/mL, and normal DUPAN-2 was defined as ≤ 150 U/mL, on the basis of standard deviations in a healthy population. Eligible patients were classified into four groups according to these upper normal limits. Among patients with normal CA19-9 levels, those with normal and high DUPAN-2 levels were categorized as normal/normal (N/N) and normal/high (N/H) groups, respectively. Among patients with high CA19-9 levels, those with normal and high DUPAN-2 levels were categorized as high/normal (H/N) and high/high (H/H) groups, respectively. CA19-9 and DUPAN-2 levels were measured simultaneously in all patients diagnosed with BTCs. Patients with jaundice underwent biliary drainage before any anticancer treatment, and tumor markers were measured after serum total bilirubin decreased to < 3.0 mg/dL. Postoperative complications were graded according to the Clavien–Dindo classification system.^11^ Overall survival (OS) was calculated from the date of surgery to the date of last follow-up or death. Recurrence-free survival (RFS) was defined as the interval from surgery to radiographically confirmed disease recurrence.

### Treatment Strategy

Standard surgical treatment for HC and ICC with hilar invasion included major hepatectomy (hemihepatectomy with caudate lobectomy or trisegmentectomy with caudate lobectomy) combined with extrahepatic bile duct resection (BDR) and bilioenteric anastomosis. The standard surgery for DC and AC consisted of pylorus-preserving pancreaticoduodenectomy (PPPD). Peripheral type ICC was treated with hepatectomy, whereas hepatopancreatoduodenectomy (HPD) was performed in cases with extensive biliary tract invasion. Regional lymphadenectomy and para-aortic lymph node sampling were performed in all cases. Patients underwent computed tomography (CT) scans every 3–4 months postoperatively, and recurrence was defined as a radiographically evident tumor.

### Data Collection

The following clinical parameters were investigated and compared among the four groups: (i) clinicopathological features and (ii) OS and RFS.

### Statistical Analysis

Median values were calculated, and nonparametric statistical tests were applied. Categorical variables were compared using the chi-squared or Fisher’s exact test, as appropriate. Continuous variables were compared using the Mann–Whitney *U* test. Multiple comparisons of clinicopathological features were performed among the four groups, and combinations of groups showing significant differences were further evaluated. Deaths from any cause were included in the survival analysis. Survival curves were generated using the Kaplan–Meier method, and differences were assessed with the log-rank test. Variables significant in univariate analysis were included in multivariate analysis. The Cox proportional hazards model was used for multivariate analysis, and hazard ratios (HRs) with 95% confidence intervals (CIs) were reported. All statistical analyses were performed using JMP statistical software version 17 (SAS Institute, Cary, NC, USA) under the supervision of a statistician. Statistical significance was set at *P* < 0.05.

## Results

**Fig. 1:**
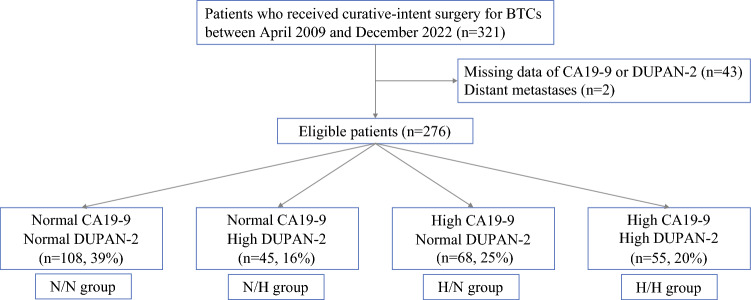
Flow diagram of the study. *CA19-9* carbohydrate antigen 19-9, *DUPAN-2* Duke pancreatic monoclonal antigen type 2, *N/N* normal CA19-9 and normal DUPAN-2, *N/H* normal CA19-9 and high DUPAN-2, *H/N* high CA19-9 and normal DUPAN-2, *H/H* high CA19-9 and high DUPAN-2

A total of 321 patients with BTC underwent curative-intent surgery between April 2009 and December 2022. Among these, 8 patients without CA19-9 measurements, 35 without DUPAN-2 measurements, and 2 with initially unresectable BTC with distant metastases were excluded. The final cohort included 276 patients, distributed as follows: N/N group (*n* = 108, 39%), N/H group (*n* = 45, 16%), H/N group (*n* = 68, 25%), and H/H group (*n* = 55, 20%). Figure 1 shows a flow diagram of study enrollment, and patient characteristics are summarized in Table 1. Among the cohort, 153 patients (55%) had CA19-9 levels ≤ 37 U/mL, and 34 (12%) had levels ≤ 2 U/mL. Clinicopathological features across the four groups are presented in Table 2. Preoperative factors, including age, sex, body mass index, and cancer subtype, did not significantly differ among the four groups. Regarding operative factors, intraoperative blood loss (mL) in the N/N group was significantly lower than in the H/N group (*P* = 0.021). The transfusion rate in the N/N group was significantly lower compared with the other three groups (*P* = 0.032, *P* = 0.044, and *P* = 0.029, respectively). Pathological analysis demonstrated that the positive rate of microvascular invasion in the N/N group was significantly lower than in the N/H and H/H groups (*P* = 0.043 and *P* = 0.039, respectively). The perineural invasion rate in the N/N group was significantly lower than in all other groups (*P* = 0.004, *P* = 0.008, and *P* = 0.005, respectively). Lymph node metastasis was less frequent in the N/N group compared with all other groups (*P* = 0.016, *P* = 0.041, and *P* = 0.009, respectively), and was lower in the H/N group compared with the H/H group (*P* = 0.036). The proportion of patients with UICC stage ≥ III was significantly lower in the N/N group compared with the other three groups (*P* = 0.014, *P* = 0.048, and *P* = 0.011, respectively).

**Table 1:** Patient characteristics (*n* = 276)

Characteristics	No. (%) or median (IQR)
Age, years	72 (66–77)
Sex, female	99 (36)
BMI (kg/m^2^)	22.3 (20.2–24.0)
Cancer type	
Intrahepatic cholangiocarcinoma	41 (15)
Hilar cholangiocarcinoma	72 (26)
Distal cholangiocarcinoma	57 (21)
Gallbladder carcinoma	53 (19)
Ampullary carcinoma	53 (19)
Operative factors	
Portal vein resection, yes	29 (11)
Arterial resection, yes	19 (7)
Operation time (min)	363 (285–750)
Blood loss (mL)	557 (301–973)
Transfusion, yes	50 (18)
Postoperative complication, grade ≥ 3	59 (21)
Adjuvant chemotherapy initiation	198 (72)
Pathological findings	
UICC pathological T factor	
T1	50 (19)
T2	107 (40)
T3	95 (35)
T4	16 (6)
Tumor differentiation, poor	28 (10)
Microvascular invasion, positive	102 (40)
Perineural invasion, positive	165 (60)
Lymph node metastases, positive	122 (44)
Surgical margin, positive	65 (24)
UICC final stage	
Ⅰ	70 (26)
Ⅱ	71 (27)
Ⅲ	100 (37)
Ⅳ	26 (10)

*IQR* interquartile range, *BMI* body mass index, *grade > 3* Clavien–Dindo classification grade > 3, *poor* poorly differentiated adenocarcinoma, *UICC* Union for International Cancer Control

**Table 2:** Comparison of patient characteristics and outcomes among the four groups

Characteristics No. (%) or median (IQR)	N/N group (*n* = 108)	N/H group (*n* = 45)	H/N group (*n* = 68)	H/H group (*n* = 55)	*P*-value
Patient characteristics					
Age, years	70 (37–86)	71 (42–86)	72 (18–89)	75 (50–87)	0.092
Sex, female	42 (39)	19 (42)	18 (30)	20 (36)	0.392
BMI (kg/m^2^)	22.8 (20.6–24.8)	22.3 (19.7–23.6)	22.8 (20.1–24.0)	21.9 (19.6–23.6)	0.339
Cancer type					0.142
Intrahepatic cholangiocarcinoma	14 (13)	7 (16)	10 (15)	10 (18)	
Hilar cholangiocarcinoma	21 (19)	11 (24)	23 (34)	17 (31)	
Distal cholangiocarcinoma	22 (20)	8 (18)	15 (22)	12 (22)	
Gallbladder carcinoma	19 (18)	9 (20)	14 (21)	11 (20)	
Ampullary carcinoma	32 (30)	10 (22)	6 (9)	5 (9)	
Operative factors					
Portal vein resection, yes	10 (9)	5 (11)	5 (8)	9 (16)	0.401
Arterial resection, yes	7 (6)	2 (4)	3 (4)	7 (13)^b^	0.352
Operation time (min)	309 (270–411)	369 (291–462)	359 (302–468)	403 (334–471)	0.053
Blood loss (mL)	514 (280–728)^a^	615 (245–1093)	744 (322–1228)^a^	660 (457–1520)	0.021
Transfusion, yes	10 (9)^b, c, d^	11 (24)^b^	14 (21)^c^	15 (27)^d^	0.016
Postoperative complication, grade ≥ 3	18 (17)	10 (22)	19 (28)	12 (22)	0.563
Adjuvant chemotherapy initiation	73 (68)	31 (69)	56 (82)	38 (69)	0.238
Pathological findings					
UICC pathological T factor					
T1–2/T3–4	70 (67)/34 (33)	21 (47)/24 (53)	43 (66)/22 (43)	23 (43)/31 (57)	0.076
Tumor differentiation, poor	11 (10)	3 (7)	6 (9)	8 (15)	0.608
Microvascular invasion, positive	29 (27)^e,f^	16 (36)	30 (44)^e^	27 (49)^f^	0.008
Perineural invasion, positive	47 (44)^g,h,i^	33 (75)^g^	46 (68)^h^	39 (71)^i^	0.006
Lymph node metastases, positive	34 (31)^j,k,l^	24 (53)^j^	32 (47)^k,m^	32 (59)^l,m^	0.016
Surgical margin, positive	20 (19)	11 (24)	14 (21)	20 (36)	0.142
UICC final stage					
Ⅰ–Ⅱ/Ⅲ–Ⅳ	73 (70)/32 (30)^n,o,p^	17 (39)/26 (61^)n^	34 (52)/31 (48)^o^	17 (32)/37 (69)^p^	< 0.001

Statistical significances between the specific groups (*P* < 0.05); ^a^N/N versus H/N; ^b^N/N versus H/N; ^c^N/N versus N/H; ^d^N/N versus H/H; ^e^N/N versus H/N; ^f^N/N versus H/H; ^g^N/N versus N/H; ^h^N/N versus H/N; ^i^N/N versus H/H; ^j^N/N versus N/H; ^k^N/N versus H/N; ^l^N/N versus H/H; ^m^H/N versus H/H; ^n^N/N versus N/H; ^o^N/N versus H/N; ^p^N/N versus H/H

*IQR* interquartile range, *N/N* normal carbohydrate antigen 19-9 (CA19-9) and normal Duke Pancreatic monoclonal antigen type 2* (*DUPAN-2), *N/H* normal CA19-9 and high DUPAN-2, *H/N* high CA19-9 and normal DUPAN-2, *H/H* high CA19-9 and high DUPAN-2, *BMI* body mass index, *UICC* Union for International Cancer Control, *grade > 3* Clavien–Dindo classification grade > 3, *poor* poorly differentiated adenocarcinoma

### Analysis of Survival

**Fig. 2:**
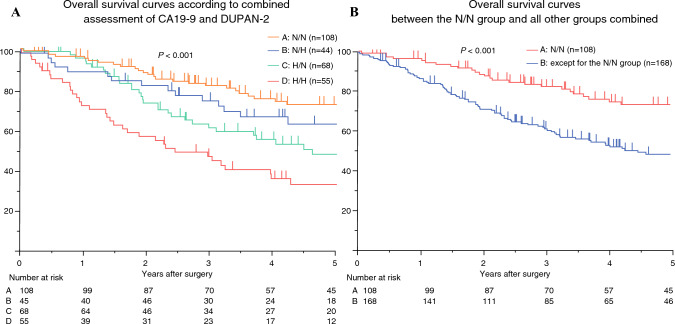
**A** Overall survival (OS) curves of patients in the N/N, N/H, H/N, and H/H groups (*n* = 276); the median OS times in the N/N, N/H, H/N, and H/H groups were not reached, not reached, 56.9 months, and 29.2 months, respectively, and the 5-year OS rates were 72.7, 60.7, 47.6, and 33.3%, respectively; **B** comparison of OS between the N/N group and all other groups combined (excluding the N/N group); the median OS for all groups except the N/N group was 51.2 months, with a 5-year OS rate of 46.9%; the N/N group had significantly better OS compared with all other groups (*P* < 0.001). *N/N* normal CA19-9 and normal DUPAN-2, *N/H* normal CA19-9 and high DUPAN-2, *H/N* high CA19-9 and normal DUPAN-2, *H/H* high CA19-9 and high DUPAN-2

**Fig. 3:**
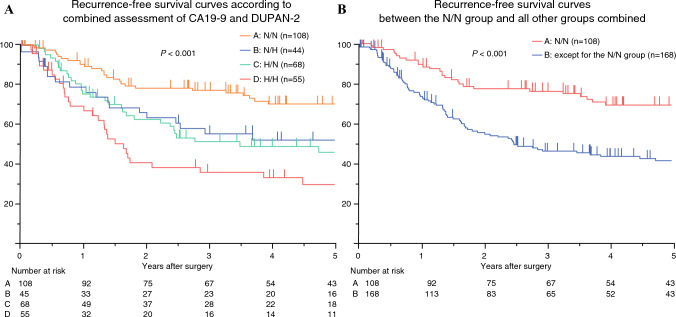
**A** Recurrence-free survival (RFS) curves of patients in the N/N, N/H, H/N, and H/H groups (*n* = 276); the median RFS times in the N/N, N/H, H/N, and H/H groups were not reached, 115.8 months, 55.3 months, and 19.6 months, respectively; and the 5-year RFS rates were 70.6, 53.5, 45.5, and 29.0%, respectively; **B** comparison of RFS between the N/N group and all other groups combined (excluding the N/N group); the median RFS for all groups except the N/N group was 34.2 months, with a 5-year RFS rate of 43.4%; the N/N group had significantly better RFS compared with all other groups *N/N* normal CA19-9 and normal DUPAN-2, *N/H* normal CA19-9 and high DUPAN-2, *H/N* high CA19-9 and normal DUPAN-2, *H/H* high CA19-9 and high DUPAN-2

The median OS for all patients was 66.7 months, with a 5-year OS rate of 58.4%. The median OS was not reached in the N/N and N/H groups, whereas it was 56.9 months in the H/N group and 29.2 months in the H/H group (Fig. 2A). The 5-year OS rates were 72.7% for N/N, 60.7% for N/H, 47.6% for H/N, and 33.3% for H/H. A stepwise decline in OS was observed across the four subgroups. The N/N group demonstrated significantly better OS compared with the H/N and H/H groups, whereas several additional pairwise comparisons did not achieve statistical significance. Detailed survival differences are illustrated in the Kaplan–Meier curves (Fig. 2A). The median OS for all groups excluding N/N was 51.2 months, with a 5-year OS rate of 46.9% (Fig. 2B), demonstrating significantly better OS in the N/N group compared with the other groups (*P* < 0.001) (Fig. 2B). The median RFS for all patients was 65.6 months, with a 5-year RFS rate of 55.7%. The median RFS was not reached in the N/N group, 115.8 months in the N/H group, 55.3 months in the H/N group, and 19.6 months in the H/H group (Fig. 3A). The 5-year RFS rates were 70.6, 53.5, 45.5, and 29.0%, respectively. A stepwise decline in RFS was observed across the subgroups. Although the N/N group showed significantly better RFS than the other groups, several pairwise comparisons among the remaining subgroups did not reach statistical significance. The detailed RFS differences are illustrated in the Kaplan–Meier curves (Fig. 3A). The median RFS for all groups excluding N/N was 34.2 months, with a 5-year RFS rate of 43.4% (Fig. 3B), showing significantly better RFS in the N/N group compared with the other groups (*P* < 0.001) (Fig. 3B). Univariate and multivariate analyses of clinicopathological factors influencing OS are presented in Table 3. Multivariate analysis identified the non-N/N status (hazard ratio [HR], 2.49; 95% confidence interval [CI], 1.61–3.89; *P* < 0.001), UICC pathological T stage ≥ 3 (HR, 2.05; 95% CI, 1.30–3.21; *P* = 0.002), and the presence of lymph node metastasis (HR, 2.44; 95% CI, 1.59–3.72; *P* < 0.001) as independent predictors of poor OS. Univariate and multivariate analyses of clinicopathological factors influencing RFS are presented in Table 4. Independent predictors of poor RFS included the non-N/N status (HR, 1.85; 95% CI, 1.19–2.89; *P* = 0.007), UICC pathological T stage ≥ 3 (HR, 1.77; 95% CI, 1.11–2.81; *P* = 0.016), and lymph node metastasis (HR, 2.55; 95% CI, 1.63–3.89; *P* < 0.001).

**Table 3:** Univariate and multivariate overall survival analyses of prognostic factors (*n* = 276)

Variables	Univariate analysis			Multivariate analysis		
	No.	MST (months)	*P*-value	HR	95% CI	*P*-value
Age, years						
< 70	107	79.7	0.647			
≥ 70	169	72.4				
Sex						
Male	177	73.7	0.555			
Female	99	104.5				
Cancer type			0.061			
Intrahepatic cholangiocarcinoma	41	57.8				
Hilar cholangiocarcinoma	72	57.5				
Distal cholangiocarcinoma	57	61.2				
Gallbladder carcinoma	53	47.9				
Ampullary carcinoma	53	Not reached				
Preoperative CA19-9 and DUPAN-2 levels						
N/N	108	Not reached	< 0.001	1.0		< 0.001
Others (N/H, H/N, and H/H)	168	51.2		2.49	1.61–3.84	
Portal vein resection						
Yes	29	25.3	< 0.001	1.49	0.88–2.72	0.187
No	247	88.3		1.0		
Arterial resection						
Yes	19	24.2	0.003	1.65	0.84–3.24	0.141
No	257	88.3		1.0		
Adjuvant chemotherapy						
Yes	198	87.2	0.005	1.71	0.92–3.12	0.097
No	78	52.7		1.0		
UICC pathological T factor						
T1 or 2	157	Not reached	< 0.001	1.0		
T3 or 4	111	37.6		2.05	1.30–3.21	0.002
Tumor differentiation						
Well or moderate	248	86.0	0.006	1.0		
Poor	28	27.2		1.76	0.98–3.11	0.051
Lymph node metastases						
Yes	122	37.7	< 0.001	2.44	1.59–3.72	< 0.001
No	154	Not reached		1.0		
Surgical margin						
Negative	211	104.5	0.001	1.0		
Positive	65	44.3		1.15	0.75–1.77	0.459
UICC final stage						
Ⅰ or Ⅱ	141	Not reached	< 0.001	1.0		0.469
Ⅲ or Ⅳ	135	38.6		1.20	0.73–1.97	

MST median survival time, *HR* hazard ratio, *CI* confidence interval, *CA19-9* carbohydrate antigen 19-9, *DUPAN-2* Duke pancreatic monoclonal antigen type 2, *N/N* normal CA19-9 and normal DUPAN-2, *N/H* normal CA19-9 and high DUPAN-2, *H/N* high CA19-9 and normal DUPAN-2, *H/H* high CA19-9 and high DUPAN-2, *UICC* Union for International Cancer Control

**Table 4:** Univariate and multivariate recurrence-free survival analyses of prognostic factors (*n* = 276)

Variables	Univariate analysis			Multivariate analysis		
	No	MST (months)	*P*-value	HR	95% CI	*P*-value
Age, years						
< 70	107	87.8	0.097			
≥ 70	169	52.4				
Sex						
Male	177	77.7	0.223			
Female	99	Not reached				
Cancer type			0.053			
Intrahepatic cholangiocarcinoma	41	42.6				
Hilar cholangiocarcinoma	72	69.4				
Distal cholangiocarcinoma	57	87.8				
Gallbladder carcinoma	53	60.7				
Ampullary carcinoma	53	Not reached				
Preoperative CA19-9 and DUPAN-2 levels						
N/N	108	Not reached	< 0.001	1.0		0.007
Others (N/H, H/N, and H/H)	168	34.2		1.85	1.19–2.89	
Portal vein resection						
Yes	29	35.2	0.136			
No	247	127.2				
Arterial resection						
Yes	19	16.3	0.002	1.83	0.98–3.40	0.164
No	257	127.2		1.0		
Adjuvant chemotherapy						
Yes	198	67.3	0.024	1.69	0.96–3.11	0.086
No	78	46.2		1.0		
UICC pathological T factor						
T1 or 2	157	Not reached	< 0.001	1.0		
T3 or 4	111	20.3		1.77	1.11–2.81	0.016
Tumor differentiation						
Well or moderate	248	127.2	0.027	1.0		
Poor	28	16.3		1.49	0.83–2.70	0.185
Lymph node metastases						
Yes	122	20.1	< 0.001	2.55	1.63–3.98	< 0.001
No	154	Not reached		1.0		
Surgical margin						
Negative	211	Not reached	0.007	1.0		
Positive	65	29.6		1.15	0.75–1.78	0.525
UICC final stage						
Ⅰ or Ⅱ	141	Not reached	< 0.001	1.0		0.924
Ⅲ or Ⅳ	135	24.1		1.02	0.60–1.74	

*MST* median survival time, *HR* hazard ratio, *CI* confidence interval, *CA19-9* carbohydrate antigen 19-9, *DUPAN-2* Duke pancreatic monoclonal antigen type 2, *N/N* normal CA19-9 and normal DUPAN-2, *N/H* normal CA19-9 and high DUPAN-2, *H/N* high CA19-9 and normal DUPAN-2, *H/H* high CA19-9 and high DUPAN-2, *UICC* Union for International Cancer Control

## Discussion

This study evaluated the prognostic impact of the combined assessment of CA19-9 and DUPAN-2 in patients with resectable BTCs. The N/N group demonstrated significantly better OS and RFS than the other groups. Multivariate analysis confirmed that normal levels of both CA19-9 and DUPAN-2 represented independent favorable prognostic factors. The combined biomarker profile may reflect underlying tumor biology and disease aggressiveness rather than a purely causal prognostic effect.

Recently, the combined assessment of CA19-9 and DUPAN-2 has shown promise in evaluating the prognosis of pancreatic cancer.^8–10^ Previous research has reported the prognostic impact of the combined assessment of CA19-9 and DUPAN-2 in patients with PDAC.^10^ In that study, the N/N group demonstrated significantly better OS compared with the other three groups, whereas OS curves for the N/H, H/N, and H/H groups were similar, with no significant differences.^10^ Furthermore, another recent study reported that the N/N group had a significantly better disease-free survival rate compared with the N/H or high CA19-9 groups.^9^ However, no studies have investigated whether the combined assessment of CA19-9 and DUPAN-2 can effectively assess prognosis in patients with BTCs. In this study, the N/N group demonstrated a significantly better prognosis. Multivariate analysis identified normal levels of both CA19-9 and DUPAN-2 as independent favorable prognostic factors. Conversely, the H/H group exhibited a significantly worse prognosis compared with the combined cohort of all other groups. These findings suggest that preoperative combined assessment of CA19-9 and DUPAN-2 may contribute to prognostic stratification of BTCs, including in patients with Lewis antigen-negative status or incomplete function of the FUT3 gene. Further investigation is warranted to determine how these biomarkers may inform neoadjuvant treatment strategies.

Previous studies have reported that aggressive pancreatic tumors actively produce Lewis a antigens to facilitate invasion and metastasis, resulting in elevated CA19-9 levels in patients with functionally normal FUT alleles. In contrast, patients with FUT3 gene deficiency (Lewis antigen-negative) or incomplete FUT3 gene function exhibit absent or markedly reduced CA19-9 production, with reductions up to 50%.^3–7^ A recent study showed that combining FUT2/FUT3 genotyping with CA19-9 and DUPAN-2 levels significantly improved the diagnosis of stage I/II PDAC. The combination guided by FUT genotyping achieved superior sensitivity and specificity for detecting stage I/II PDAC compared with CA19-9 alone or an unstratified combination. Notably, even without FUT stratification, combining CA19-9 and DUPAN-2 increased sensitivity (82.8 versus 68.0%) while maintaining comparable specificity (91.3 versus 92.9%). Furthermore, the area under the curve also increased from 0.839 to 0.935, indicating improved diagnostic accuracy for stage I/II PDAC.^7^

The rates of perineural invasion and lymph node metastasis were significantly lower in the N/N group compared with the remaining groups. Additionally, the rate of microvascular invasion was significantly lower in the N/N group than in the N/H and H/H groups. These findings suggest that the preoperative combined assessment of CA19-9 and DUPAN-2 may aid in predicting advanced disease, including lymph node metastasis. Moreover, lymph node metastasis was identified as an independent predictor of poor prognosis in this study, consistent with previous reports.^12–14^ Thus, predicting lymph node status before curative resection or treatment initiation is crucial. Several studies have reported preoperative imaging parameters, including CT, magnetic resonance imaging, and F-18 fluorodeoxyglucose positron emission tomography (FDG–PET), for predicting lymph node metastasis.^15–18^ However, the preoperative determination of lymph node metastasis status using imaging remains challenging in patients with BTCs.

This study has several limitations. First, the analysis was based on a single-center database, and the possibility of unexpected bias cannot be entirely excluded. Second, some patients were excluded because DUPAN-2 and/or CA19-9 levels were not measured. Third, although serum levels of CA19-9 and DUPAN-2 were measured after biliary drainage and normalization of total bilirubin levels (< 3.0 mg/dL) as previously reported,^10,19^ a residual influence of prior obstructive jaundice may not have been completely excluded. Therefore, the interpretation of tumor marker values requires caution, given the potential residual effect of obstructive jaundice. Fourth, the number of patients in each cancer subtype group was relatively small. Fifth, the study included only patients who underwent radical surgery and excluded those with unresectable BTCs; intention-to-treat analysis was not performed. Sixth, although this study focused on BTCs, these malignancies represent a heterogeneous group of tumors with distinct biological characteristics across subtypes, and subtype-specific analyses were not feasible owing to the limited sample size within each subgroup. Seventh, besides tumor markers, modalities such as FDG–PET, circulating tumor deoxyribonucleic acid, and germline analysis may serve as useful tools for preoperative evaluation in the future. Eighth, the FUT3 function was not directly assessed in this study. Additionally, surrogate markers of FUT3 activity, aside from the combined CA19-9 and DUPAN-2 assessment, were not evaluated. Finally, given the retrospective design, the prognostic value of the combined biomarker assessment may reflect underlying tumor biology and disease aggressiveness. Therefore, prospective studies with larger sample sizes conducted across multiple institutions are warranted.

In conclusion, normal levels of both CA19-9 and DUPAN-2 were independent favorable prognostic factors. Moreover, preoperative combined assessment of CA19-9 and DUPAN-2 may contribute to prognostic stratification of BTCs, including in patients with Lewis antigen-negative status or incomplete function of the FUT3 gene.
